# Single-Multiplex Detection of Organ Injury Biomarkers using SPRi based Nano-Immunosensor

**DOI:** 10.1038/srep36348

**Published:** 2016-10-31

**Authors:** Effat Zeidan, Siqi Li, Zhiguo Zhou, Jennifer Miller, Marinella G. Sandros

**Affiliations:** 1 Department of Nanoscience, University of North Carolina at Greensboro, Greensboro, NC 27455, USA; 2 Luna Innovations Incorporated, Danville, VA 24541, USA; 3 HORIBA Scientific, Edison, NJ 08820, USA

## Abstract

The clinical assessment of multiple organ dysfunctions at early stages is recognized to be an important factor in prompting definitive treatment decisions that prevent irreversible organ damage. In this article, we propose a real-time, label-free, and multiplex nanoenhanced SPRi platform to quantitatively assess two biomarkers, kidney injury molecule (KIM-1) and high mobility group box-1 (HMGB-1) simultaneously in buffer. Our work involves three major contributions in the design of the immunosensor: (1) we applied site-specific immobilization of antibodies to the solid surface that avoids loss of biological activity caused by covalent attachment; (2) we constructed a well-blocked sensor surface that exhibits minimal non-specific adsorption for singleplex measurements of each biomarker in buffer; and (3) we adopted a sandwich assay that implements functionalized quantum dots (NanoEnhancers) as signal amplifiers to achieve a sensitivity level of 5 pg/mL for KIM-1 and HMGB-1 in buffer. We foresee great potential and success in extending this multiplex and ultra-sensitive platform to assess a variety of other emerging clinical biomarkers at low concentrations and in complex matrices.

Despite that recent advances in targeted therapy and surgical care have caused a drastic decrease in mortality rates, irreversible organ failure persists as the leading cause of high morbidity in critically ill patients^1^,^2^. In most cases, the curative strategies fail to halt organ disease progression before reaching an irreversible stage due to a delay in the decision to begin treatment. The medical intervention decision highly depends on the ability of clinical tests to detect indicative biomarkers in bodily fluids. The development of such sensitive and non-invasive clinical assays not only will allow medical treatment at early stages; but also will enhance curative strategies, improve the quality of life and provide better insight onto the mechanistic basis of organ injury.

Currently, many analytical techniques with varying sensitivity and specificity have been developed and employed to detect clinical biomarkers; however, they are very limited in predicting disease progression at early stages. Mass spectrometry-based tools for example 2D/MALDI-MS and LC-MS/MS suffer from low sensitivity and require intensive data analysis done by professionals^3^. Immunoassays on the other hand such as enzyme linked immunosorbent assay (ELISA) are labor intensive, require frequent calibration, and are incapable of acquiring data in a high-throughput manner^4^. Nucleic acid based assays not only require a complex hardware setup but also suffer from interference in some matrix types^5^,^6^. Finally, in most of these assays, the degree of accuracy highly depends on the expertise of the pathologist. Hence, there is a lack of diagnostic tools that could accurately detect a panel of clinical biomarkers at the earliest time point possible and in an ease of use manner. This gap in clinical diagnostic technology is the major impediment to treating organ injuries in the early stages.

In the present work, an *in vitro* diagnostic optical assay based on surface plasmon resonance imaging (SPRi) is used to overcome the drawbacks of currently used tests to sensitively assess individual or multiple organ injury biomarkers at the same time. The presented platform is still in its early stages and requires further development for proper implementation in clinical setting. SPRi is a non-labeling and real time optical technique for the detection and analysis of biomolecular interactions at the surface of a high refractive index glass prism coated with a thin layer of metal. The sensing mechanism measures changes in refractive index up to ~300 nm from the metal surface. The surface plasmons are sensitive to refractive index changes occurring in the vicinity of the metal layer; hence, serving as the basis of the detection of biomolecular interactions.

Since the SPRi microarray platform allows for the quantitative detection of multiple interactions simultaneously better known as multiplexing, many studies have focused on this promising application to screen a variety of analytes in different type of matrices. For example, one group responded to the need of screening antimicrobial drug residues in milk due to their possible health risks by developing a competitive immunoassay for the simultaneous detection of seven drug residues down to ppb levels^7^. Others have extended multiplex sensing to low molecular weight protein biomarkers with clinical significance in the body, such as ß_2_-microglobulin (MW = 11.8 kDa) and cystatin C (MW = 13.4 KDa) to reach nM limit of detection (LOD)^8^. Multiple inflammatory cytokines (IL-1, IL-6, and TNF-α) were also detected in saline buffer and cell culture medium at ng/mL levels to better serve in understanding disease diagnosis and progression^9^. Moreover, the parallel detection of cancer biomarkers in buffer and in blood samples has attracted much interest in a number of studies leading to the construction of robust and low-fouling assays with good sensitivity to improve current diagnostic tests^10^,^11^. Therefore, excellent potential has been demonstrated by the novel list of microarray biosensors developed; however, sensitivity and reproducibility are key characteristics that still demand improvements to meet the needs for clinical diagnostic analysis. Developing novel immobilization techniques that avoid alterations to the functionalities of biological receptors and adopting signal amplifiers to decrease the LOD of biomarkers represent the areas where improvement can be applied for enhanced reproducibility and performance.

Hence, the work presented here introduces a dual microarray platform for the multiplex and ultrasensitive detection of two biomarkers, the High Mobility Group Box 1 (HMGB-1) and Kidney Injury Molecule-1 (KIM-1) biomarkers, which have shown potential prognostic value in acute liver disease^12^,^13^,^14^ and acute renal failure respectively^15^. This platform incorporates specific capture antibodies for both biomarkers on the surface and NanoEnhancers-labeled detection antibodies to enable the ultrasensitive detection of biomarkers at 5 pg/mL (200 fM) concentrations. From our previous work, improving the sensitivity of SPRi was achieved using NanoEnhancers independent of the type of capture ligand or analyte and even environmental conditions^16^,^17^. Ultimately, the early detection of these biomarkers could provide detailed information on the disease prognosis and assist in refining patient care.

## Results

### Biosensor surface optimization

**Figure 1:**
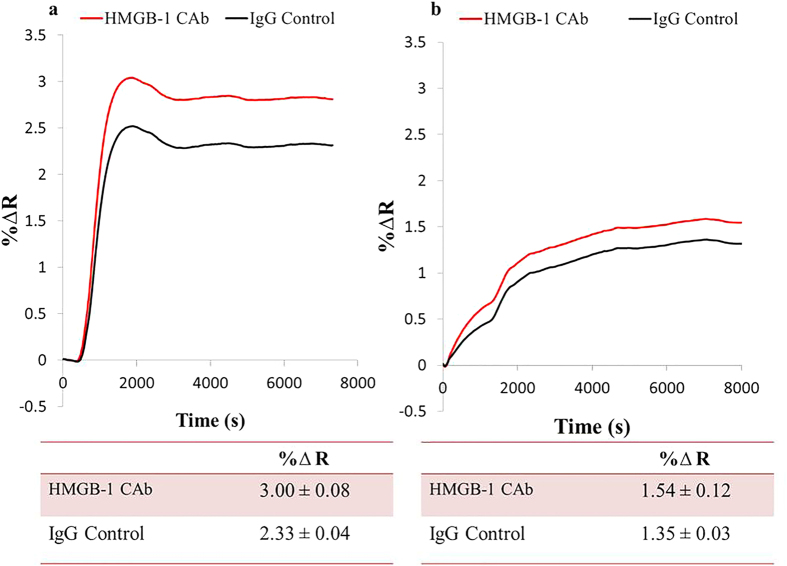
Comparative biosensor behavior in response to HMGB-1 (4 μg/mL) in buffer. Both experiments A and B were carried out under the same conditions and prepared using the alkanethiol/EDC/NHS surface onto which the HMGB-1 monoclonal capture antibodies were covalently bound (amine coupling). Experiment (**a**) resulted in a low specificity signal of ΔR = 3% ± 0.08; while experiment (**b**) gave a lower and inconsistent response of ΔR = 1.5% ± 0.12.

The overall performance of the SPRi biosensor highly depends on the quality of the surface functionalization and the proper anchoring of the biorecognition probes onto the metal surface. Most gold surfaces are functionalized by alkanethiol organic compounds as the primary building blocks onto which the biological ligands are covalently bound. For example, the immobilization of antibodies in immunoassays can be performed by the amine coupling of lysine residues in antibodies to the surface linked carboxylic acid groups through an EDC/NHS intermediate activation step. This surface chemistry has been widely used and successful in many previous works^18^,^19^,^20^; however, we found this strategy not to be suitable for our specific experimental setup. It resulted in inconsistency of surface uniformity from one experiment to the other as shown in Fig. 1. Therefore, to address this issue and survey to the different configurations of ligands introduced in this study, we chose to design the capture array by immobilizing the KIM-1 (50 μg/mL) and HMGB-1 (50 μg/mL) monoclonal capture antibodies onto different areas of protein A coated surface to avoid structural alterations to the antibodies, ensure proper orientation of the antibody and uniform coverage on the surface^21^. We found that coating the surface with protein A (5 μg/mL) solution for 4 hours enabled a homogeneous coverage and active binding of the capture antibodies spotted on the surface, which enhanced the sensitivity and reproducibility of the SPRi response. In addition, 50 μg/mL spotting concentration of both HMGB-1 and KIM-1 monoclonal antibodies was used for all the SPRi measurements since this specific surface coverage produced the highest positive SPRi signal upon the interaction of the antibodies with the specific analyte. The relative concentration of each spot on the sensor surface is well controlled by using a robotic microarrayer, which consistently prints the antibody ligands onto the sensor surface of each experiment. Moreover, the SPRi signal obtained from the array of antibody spots (N = 4) is averaged and reported. The same procedure of immobilization of the monoclonal antibodies (KIM-1 and HMGB-1) and SPRi measurements for both EDC/NHS and protein A sensor surfaces was performed; however, the main difference was the chemistry of the binding of the monoclonal antibodies to the sensor surface. Therefore, protein A surface chemistry was adopted for all remaining experiments due to the reproducibility and reliability of the immunosensor.

### Minimizing non-specific interactions

**Figure 2:**
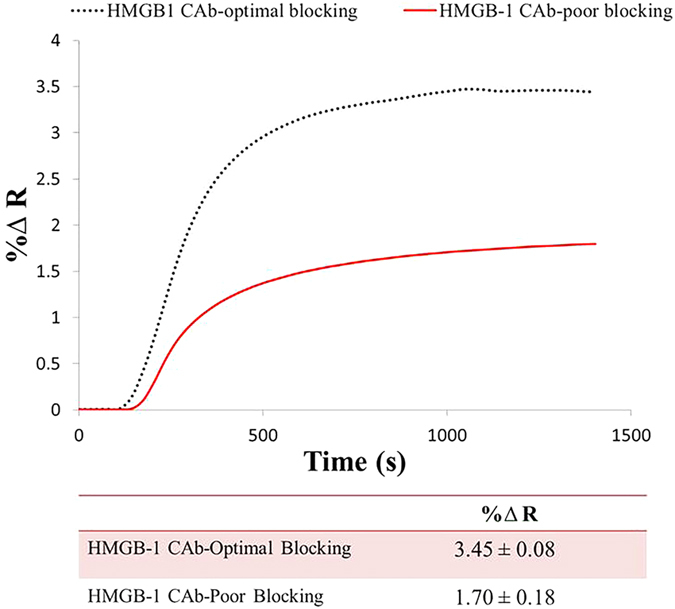
Comparative SPRi sensorgrams to HMGB-1 binding to two surfaces under different blocking conditions. Above figure represents the binding kinetics of HMGB-1 (50 ng/mL) to two surfaces functionalized with HMGB-1 capture antibodies (50 μg/mL) and blocked under two conditions. The first surface was blocked using a combination of 0.1 mM PEG2000 and 1% BSA resulting in a signal of 1.7% ΔR, ±0.18% (red solid line). While the second surface was blocked using a combination of 2 mM PEG2000 and 1% BSA and the binding of HMGB-1 resulted in a signal of 3.45% ΔR, ±0.08% (black dotted line).

A well-designed immunoassay sensor not only relies on the proper immobilization of the biological ligands but also on the suppression of non-specific adsorption of analyte on the metal surface. And the challenge lies in choosing a blocking agent of suitable dimensions and properties that can minimize the potential of non-specific signal but at the same time allow accessibility to the binding site of the bioreceptor. In this study, we found that the sequential blocking using 2 mM polyethylene glycol (PEG2000) and 1% bovine serum albumin (BSA) provided optimal coverage of the surface against non-specific adsorption of analyte. The concentrations used were critical as increasing the concentration of PEG2000 from 0.1 mM to 2 mM while keeping the BSA concentration at 1% as well blocking the surface with proper sequence, enhanced the signal significantly from (1.7% ΔR, ±0.18%) to (3.45% ΔR, ±0.08%), as shown in Fig. 2. Higher concentrations of PEG2000 (>2 mM) did not result in enhanced SPRi signals, which entails that the surface is well blocked and saturated at this concentration. Similarly, 1% BSA was chosen as the saturation limit for the sensor surface at which optimal SPRi signal was obtained.

In addition, the protein A coated surface was further blocked by fc fragments (0.5 μg/mL) after injection of biomarker in order to occupy the free protein A regions not bound to the immobilized antibodies. Hence, with the combination of multiple agents, we were able to reach a negligible non-specific binding signal allowing our platform to perform successfully in the multistep sensing mechanism.

### Singleplex measurements of KIM-1 and HMGB-1

A sandwich assay was used to detect KIM-1 and HMGB-1 separately at low concentrations in buffer. The capture array was first coated with protein A (5 μg/mL, 4 hours) and then monoclonal KIM-1 and HMGB-1 antibodies and rabbit monoclonal IgG antibodies (negative control) were directly attached to the surface at 50 μg/mL spotting concentration. The functionalized biosensing surface was then blocked using a combination of BSA (1%) and PEG2000 (2 mM) inside the instrument. The detection of biomarkers was accomplished in a three-step process.

At first, the biomarker was injected and followed by sequential injection of the fc fragment to block the unoccupied protein A bound to the sensing surface. Secondly, specific biotinylated KIM-1 detection antibodies were injected to bind to the captured analyte. Finally, the signal amplification was accomplished reproducibly with a non-interfering streptavidin coated NanoEnhancers. The streptavidin coated NIR quantum dots are conjugated to one or more biotinylated secondary antibodies specific to HMGB-1 and KIM-1 analytes. Therefore, one NIR quantum dot can bind to one analyte or more on the surface. The introduction of NIR quantum dots in the amplification process is known to lead to signal amplification due to the mass loading effect of the nanoparticles and possible emission coupling with the oscillating surface plasmons on the metallic surface^16^.

**Figure 3:**
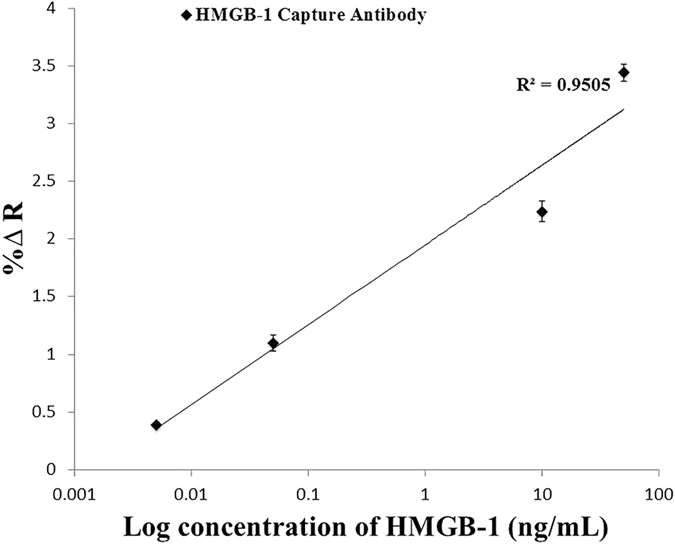
A concentration profile of Nanoenhanced SPRi signal in response to HMGB-1 in buffer. SPRi response (%∆ Reflectivity) represents the NanoEnhanced response upon the binding of NanoEnhancers to the various concentrations of HMGB-1 biomarker (ng/mL) in phosphate buffer. The % change in reflectivity reported takes into account the IgG negative control signal, which is subtracted from the overall signal.

**Figure 4:**
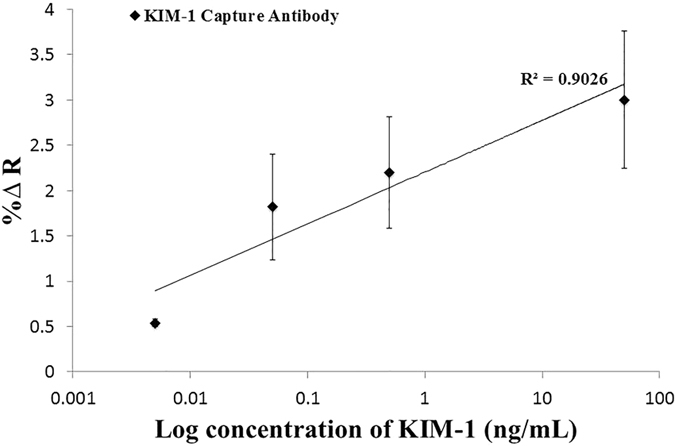
A concentration profile of Nanoenhanced SPRi signal in response to KIM-1 in buffer. SPRi response (%∆ Reflectivity) represents the NanoEnhanced response upon the binding of NanoEnhancers to the various concentrations of KIM-1 biomarker (ng/mL) in phosphate buffer. The % change in reflectivity reported takes into account the IgG negative control signal, which is subtracted from the overall signal.

A concentration range (0.005, 0.05, 10, and 50 ng/mL) of HMGB-1 biomarker in phosphate buffer was detected and the specific SPRi responses obtained are presented in (Fig. 3). Secondly, a concentration range (0.005, 0.05, 0.5, and 50 ng/mL) of KIM-1 biomarker was tested and the results of the SPRi response are shown in (Fig. 4). The results presented in Figs 3 and 4 represent the plot of the average SPRi signal versus the log of the concentration of HMGB-1 and KIM-1 analyte-antibody binding signal. This plot is fit to a linear regression line, which includes data points with error bars representing the standard deviation of the analyte-antibody binding signal obtained from multiple experiments. From these plots we were able to obtain the LOD for both biomarkers to be 5 pg/mL. The LOD represents the minimum detectable concentration for which the SPRi signal has a 3-fold higher response than the negative control taking into consideration the standard error. Lower concentrations than the LOD are not presented because the response obtained is comparable to the negative control; therefore, resulting in a null normalized SPRi response.

### Multiplex measurements of both KIM-1 and HMGB-1

To further investigate the multi-target potential of this biosensing platform, both KIM-1 and HMGB-1 antibodies along with the negative rabbit IgG control were spotted on the same surface at 50 μg/mL concentrations. The SPRi apparatus used to perform the assay measurements, incorporated HORIBA software which allows the user to select and define the specific antibody ligand spots printed on the surface; therefore, distinctively separating the SPRi signals obtained from each ligand of interest. The results presented represent the average signal obtained from multiple ligand spots corresponding to various experiments.

**Figure 5:**
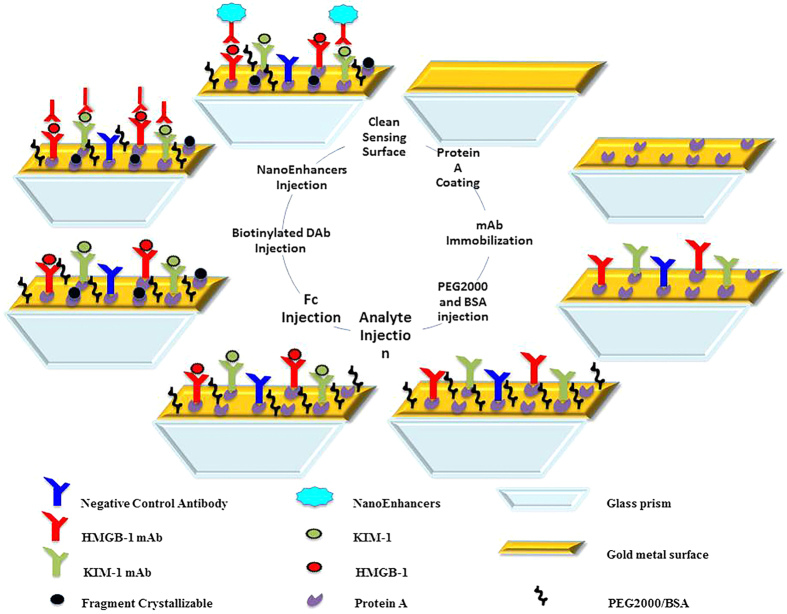
The step-wise experimental approach in the design of sandwich assay. The setup demonstrates the sequential steps performed to design the immunosensor surface as well as to carry out the multiplex measurements of both KIM-1 and HMGB-1 biomarkers. Firstly, a clean surface is coated with protein A, followed by the immobilization of monoclonal antibodies, and then the surface is blocked with a combination of PEG2000 and BSA. The multiplex measurements are carried out by the sequential injection of biomarker, fc, biotinylated secondary antibody and finally NanoEnhancers to amplify the signal.

The multiplex detection involved a three-step sandwich assay process very similar to the individual biomarker detection previously presented. The first step involved the injection of both biomarkers in buffer followed by injection of fc fragment (0.5 μg/mL) to further block the unoccupied protein A regions. The second step introduced the biotinylated KIM-1 detection antibody and finally the NIR streptavidin-coated QDs were injected as the amplification probes (Fig. 5).

**Figure 6:**
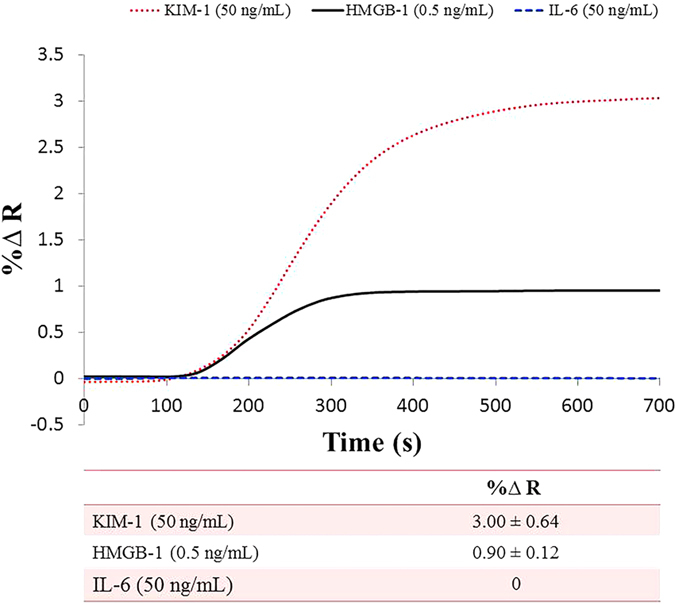
Multiplex SPRi sensorgram of KIM-1, HMGB-1 and IL-6 control. The sensorgram represents the Nanoenhanced SPRi response (%∆ Reflectivity) versus time (s) of 50 ng/mL KIM-1 biomarker (dotted red line), 0.5 ng/mL) HMGB-1 biomarker (solid black line), and 50 ng/mL IL-6 control (dashed blue line) measured in phosphate buffer.

The results of the multiplex sandwich-amplification assay that detects HMGB-1 (0.5 ng/mL), KIM-1 (50 ng/mL) and IL-6 negative control (50 ng/mL) are presented in Fig. 6. Different analyte concentrations were used in order to demonstrate the multiplex potential of the immunosensor. The SPRi response is highest for the KIM-1 biomarker (~3% ΔR, ±0.64), and lower for the HMGB-1 biomarker (~0.9% ΔR, ±0.12), with a negligible signal for the IL-6 negative control.

## Discussion

We adopted a non-covalent attachment setup to immobilize the capture antibodies onto the sensing surface in order to avoid inconsistent results and enhance the reproducibility of the assay^21^. The inconsistency resulting from the chemical attachment of antibodies could perhaps be caused by the disordered orientation of the capture probes on the surface, which in turn could lead to loss of biological activity and hindrance in the analyte accessibility onto the binding site^22^. Protein A was bound to the sensing surface as the primary building block through the thiol-gold bond onto which the antibodies will be spotted. Protein A is mainly used in antibody-purification columns as it binds to the fragment crystallizable (Fc) region of the antibody leaving the antibody-antigen binding site (Fab) more accessible to bind to the biomolecule. An optimal density of protein A (5 μg/mL) and a spotting concentration of the capture antibodies (50 μg/mL) were utilized in the platform to ensure exposure of the binding site of the antibody and a sterically favored distance between the assembled antibodies. As a result, the sensing capability of the assay was enhanced and showed good reproducibility.

Moreover, functionalizing the surface with non-fouling materials took many attempts prior to finding the proper combination as well as concentration. It is important to note that this surface design allows more exposure to the bare gold metal surface than surfaces covered with alkanethiol groups in the layer below the antibodies. Therefore, blocking with PEG2000 chains allowed (2 mM) coverage mainly of the areas between the spotted antibodies through the thiol-gold bond as well as the rest of the sensor chip. Additionally, blocking with BSA (1%) was performed to offer extra protection as a coating layer to the protein A regions that were not occupied with antibodies. Since this surface setup is used for the first time in multiplex sandwich-amplification assay, a further non-specific adsorption challenge arose from the possible binding of the biotinylated complementary detection antibodies to the protein A. Therefore; to avoid this problem Fc was injected to occupy the remaining unoccupied protein A sites. Furthermore, the injection occurred after the injection of the analyte in order to prevent any steric hindrance that fc might cause to the binding site of the capture antibodies.

Finally, with all the surface development steps accomplished, singleplex detection for both HMGB-1 (~29 kDa) and KIM-1 (30 kDa) was performed in a sandwich-amplification assay achieving a limit of detection of 5 pg/mL (200 fM) for both biomarkers. This high level of sensitivity was accomplished by the addition of the NanoEnhancers resulting in superior sensitivity and LODs compared to reported values from currently exploited detection tools as these biomarkers are at very low clinical concentrations (ng/mL) at early stages^23^. For example, a LOD of 10 ng/mL in serum for HMGB-1 was achieved utilizing a combination of aptamer nanotechnology and electrophoresis^24^, and a LOD of 2 ng/mL in an ELISA assay performed to selectively detect HMGB-1 in serum^25^. On the other hand, KIM-1 was detected using an immunochromatographic assay at concentrations higher than 800 pg/mL^26^. A further contribution to obtain more insight on disease detection and progression is accomplished by the multiplex sandwich-amplification assay that detects HMGB-1 (0.5 ng/mL) and KIM-1 (50 ng/mL) simultaneously to yield a higher signal for the KIM-1 biomarker (~3% ΔR), a lower signal corresponding to the HMGB-1 biomarker (~0.9% ΔR) as expected, and a negligible signal for the IL-6 negative control. From these results, we can conclude that the platform demonstrates specificity and selectivity.

The enhancement in SPRi signal caused by the addition of the NanoEnhancers to the sensing surface could perhaps be caused by multiple reasons. The NanoEnhancers are heavy metal nanoparticles that result in a high mass loading effect responsible for the increased SPRi response. In addition, the NIR quantum dots possess unique quantum properties, which could possibly allow energy coupling with the gold metal layer on the surface as reported in one previous study^16^. Lower limits of detection have been reported by this previous study, which utilized the same NanoEnhancers; however, the capture ligand used was DNA whereas in this study, monoclonal antibodies are used and there is a considerable size difference between the two. For example, the size range of antibodies is about 10 nm; whereas, DNA size is about 1–2 nm. Since the SPR signal enhancement by the NanoEnhancers is distance dependent, then the closer the NanoEnhancers are to the sensor surface, the higher the signal amplification.

In future work, the proposed platform can be further investigated for the analysis of a vast number of different biomarkers simultaneously and in a variety of complex matrices. Furthermore, potential adjustments to the platform can advance the multiplex effectiveness of the technique to not only assess biological molecules and organ injury biomarkers; but also environmental toxins, microbes, drugs and many more. Therefore, we anticipate great contribution from the practical capabilities of this platform to medical, pharmaceutical and environmental research.

## Methods

### Chemicals and materials

Bare gold biochips were purchased from HORIBA Scientific. Nanostrip was purchased from Cyantek (CA, USA). Protein A was purchased from Protein Mods (WI, USA). KIM1 and HMGB1 capture antibodies were purchased from Abnova (Taipei, Taiwan). Biotinylated HMGB1 detection antibody was purchased from Abnova and KIM1 detection antibody was also purchased from Abnova and was functionalized with biotin using the biotinylation kit purchased from VWR (PA, USA). The fc fragment was purchased from EMD Millipore (MA, USA). Rabbit IgG polyclonal antibodies, polyethylene glycol (PEG2000), and bovine serum albumin (BSA) were purchased from Sigma Aldrich (PA, USA). Phosphate buffered saline (PBS) tablets and D (+)-Sucrose were purchased from fisher scientific (PA, USA). Streptavidin coated near infrared quantum dots (NIR-QDs) were purchased from life technologies (NY, USA).

### Sensor surface preparation

The bare gold biochip was cleaned using a nanostrip solution (under sonication for 90 mins, 50 °C) and then rinsed thoroughly with deionized water, dried with a stream of Nitrogen and placed in the UV/Ozone for 5 mins prior to surface functionalization. Protein A (5 μg/mL) was immobilized on the surface of the biochip and incubated for 4 hours at room temperature and a relative humidity of ~75%. Following this step, capture antibodies specific for KIM-1 (50 μg/mL), HMGB-1 (50 μg/mL) as well as Rabbit IgG antibodies (50 μg/mL, negative control) were deposited on the surface (500 μm spots) using a LabNext Microarrayer system. This spotting system prints the antibodies in an orderly and systematic manner on the sensor chip using a 500 μm Teflon pin. It is important to note that both KIM-1 and HMGB-1 antibodies along with the negative control were attached to the surface in the multi-target sensor surface design; however, only one type of antibody with the negative control was immobilized in the individual biomarker detection measurements to determine the limit of detection.

The biochip was then allowed a 2-hour incubation time for efficient immobilization at room temperature and relative humidity of 75%. Prior to SPRi measurements, the functionalized surface was blocked inside the instrument using a combination of PEG2000 (2 mM) and BSA (1%) sequentially injected above the sensor surface.

### SPRi measurements

The analysis of biomolecular interactions of the different capture ligands with the corresponding biomarkers was performed in a Horiba SPRi-PlexII model (Horiba Scientific, France) designed particularly for multiplexing purposes. The biochip was loaded into the hexagonal flow cell (11 μL capacity) inside the instrument and a running buffer (10 mM PBS, PH = 7.4), controlled at a rate of 20 μL/min by a continuous flow syringe pump (Harvard Apparatus PHD 2000 Infusion), was flown over the surface. Specific spots of each capture antibody (24 spots, 400 μm diameter each) were selected and defined using the SPRi view software. The sensing mechanism utilized a high stability LED incident light source that hits the glass prism coated with 5 nm Chromium adhesion layer and 50 nm gold, a detector that collects the resulting reflected light and a CCD camera that provides a real-time digital contrast image of the sensing surface. This image component reflects the biomolecular binding events happening at the metal surface; hence, confirming the changes in reflectivity in the SPRi response. Bright areas on the surface represent a binding event and dark spots are vice versa.

Prior to kinetic monitoring, the surface was sequentially blocked by the injection of PEG2000 (2 mM) and BSA (1%) prepared in the running buffer. This was followed by a calibration procedure involving the injection of sucrose (3 mg/mL) above the surface to account for buffer changes in all ligand spots on the surface.

The flow rate was then slowed down to 10 μL/min and allowed to equilibrate prior to the interaction measurements. This was done to permit an increased interaction time between the biomarker and the capture antibody until the detection and amplification probe are introduced to the surface.

The kinetics of the biomolecular interactions was monitored through the sequential injection of the biomarkers, fc fragment, biotinylated detection antibodies and finally the NIR-QDs for signal amplification. Each injected sample was prepared in the running buffer at room temperature. The fc fragment was introduced after the biomarkers to avoid any possible steric hindrance blocking the antibody-binding site from reacting with the biomarker of interest. The introduction of biotinylated detection antibodies served to bind to the analyte on one hand and to the streptavidin coated NIR QDs through the strong biotin/avidin non-covalent interaction on the other hand.

The SPRi response reported is the average response of 4 spots of each capture antibody on the surface with the non-specific (negative control) signal subtracted.

## Additional Information

**How to cite this article**: Zeidan, E. *et al*. Single-Multiplex Detection of Organ Injury Biomarkers using SPRi based Nano-Immunosensor. *Sci. Rep.* **6**, 36348; doi: 10.1038/srep36348 (2016).

**Publisher’s note:** Springer Nature remains neutral with regard to jurisdictional claims in published maps and institutional affiliations.
